# Correlation between tumor growth rate and survival in patients with metastatic breast cancer treated with trastuzumab deruxtecan

**DOI:** 10.1093/oncolo/oyaf057

**Published:** 2025-05-11

**Authors:** Philip He, Dhiraj Gambhire, Haiming Zhou, Xiaoyang Ma, Yoshihiro Emura, Abderrahmane Laadem, David Leung, Susan Bates, Antonio Tito Fojo, Olivier Rixe

**Affiliations:** Daiichi Sankyo Inc., Basking Ridge, NJ, United States; Daiichi Sankyo Inc., Basking Ridge, NJ, United States; Daiichi Sankyo Inc., Basking Ridge, NJ, United States; Daiichi Sankyo Inc., Basking Ridge, NJ, United States; Daiichi Sankyo Inc., Basking Ridge, NJ, United States; Daiichi Sankyo Inc., Basking Ridge, NJ, United States; Daiichi Sankyo Inc., Basking Ridge, NJ, United States; Herbert Irving Comprehensive Cancer Center, Columbia University, New York, NY, United States; James J Peters VA Medical Center, Bronx, NY, United States; Herbert Irving Comprehensive Cancer Center, Columbia University, New York, NY, United States; James J Peters VA Medical Center, Bronx, NY, United States; Daiichi Sankyo Inc., Basking Ridge, NJ, United States

**Keywords:** metastatic breast cancer, tumor growth rate, biomarkers, intermediate endpoint

## Abstract

**Background:**

Previous studies in multiple metastatic tumors treated with diverse anticancer agents including immunotherapy, chemotherapy, mAb, and TKIs have suggested the rate of tumor growth (g‐score) is inversely associated with survival.

**Methods:**

We performed a retrospective analysis of patients with metastatic breast cancer (mBC) treated with trastuzumab deruxtecan (T‐DXd), ado‐trastuzumab emtansine (T‐DM1), or chemotherapy to investigate the impact of those therapies on g‐score and explore the association of g‐score with clinical outcomes. This is the first report assessing g‐score in tumors treated with an ADC.

**Results:**

We investigated the association of g‐score with progression‐free (PFS) and overall survival (OS) in 2 phase 3 studies in patients with HER2 + mBC (DESTINY‐Breast03 (DB-03)) and HER2‐low mBC (DESTINY‐Breast04 (DB-04)). After grouping patients according to quartiles of g‐scores, we explored the association between g‐score and PFS/OS using Kaplan‐Meier plots and Cox regression models. The median g‐score was higher for T‐DM1, suggesting a faster growth rate at 0.0009/day vs that for T‐DXd at 0.0002/day (*P* < .0001). Additionally, with data collection stopped at the time of database lock, 23% and 48% of tumors demonstrated only regression without growth in the T‐DM1 and T‐DXd arms, respectively. In DB-04, median g was 0.0018/day and 0.0006/day (*P* < .0001); with 17% and 32% of tumors demonstrating only regression with treatment of physician’s choice (TPC) and T‐DXd, respectively.

**Conclusions:**

Compared to T‐DM1 and TPC therapies, T‐DXd significantly reduced the rate of tumor growth in the overall population and across subgroups. In both studies, the tumor growth rate was inversely associated with PFS and OS. In addition, it showed improved concordance with survival compared to ORR. The use of tumor growth rate as an intermediate endpoint may potentially accelerate drug development and reduce a patient’s exposure to agents with limited or no activity.

**Implications for practice**

Tumor growth rate (g-score) may significantly impact the way of conducting clinical trials. The use of tumor growth rate as an intermediate endpoint with a limited patient sample may potentially accelerate drug development and patient access to relevant new therapeutics (herein ADCs) through accelerated approval or prioritized phase 3 clinical trials. By contrast, it may reduce patient’s exposure to agents with limited (or no) meaningful activity and prevent the conduction of large, randomized trials with a low probability of success. Current daily practice for treatment evaluation is based on tumor shrinkage. Continued research is needed to evaluate whether an individual patient may benefit from tumor growth rate determined by a limited number of CT-scans, compared to relevant benchmarks in similar populations, to better guide standard of care therapy in the metastatic setting.

## Introduction

Despite the various mechanisms established by regulatory agencies in expediting drug development and approvals such as breakthrough designation, accelerated approval in the United States, and conditional approval in Europe, oncology drug development still takes a long time, on average 6‐12 years, from discovery to approval in advanced malignancies.^1^ Late‐stage clinical trials still suffer relatively high failure rates such as in brain tumors^2,3^ and advanced pancreatic cancer.^4^ To change this situation and make drug development more efficient and less costly, it is essential to effectively translate the scientific advances from discovery to the clinic and make informed decisions based on clinical data and state‐of‐the‐art analytic tools. Overall survival (OS) is considered as the gold standard for benefit-risk assessment, particularly in the metastatic setting.^5-7^ In early‐phase oncology clinical trials, the objective is to detect a preliminary efficacy signal with a limited number of subjects treated with an experimental therapy.^8^ Thus, it becomes crucial to have intermediate or surrogate endpoints that can be measured early and that are also reasonably likely to predict long‐term survival outcomes in order to make well‐informed Go/No‐Go decisions. A novel paradigm^9^ was proposed for predicting drug efficacy using a derived growth rate (g‐score) based on serial prostate‐specific antigen (PSA) values and the approach was also applied to multiple cancers based on tumor burden assessed by radiographic measurements. The association of g‐score with survival has been explored in breast cancer,^10,11^ colorectal cancer,^12,13^ neuroendocrine,^14^ NSCLC,^15^ ovarian cancer,^16^ pancreatic cancer,^4^ prostate cancer,^9,17-22^ and renal cell cancer^23-26^ with a full range of anticancer agents including chemotherapy, immunotherapy, and targeted hormonal and monoclonal antibody therapy. This paradigm is based on a model that assumes changes in tumor or PSA quantity resulted from two independent but simultaneous processes—exponential decay or regression of the tumor at rate d and exponential growth or re-growth at rate *g*. Compared to traditional tumor assessment such as response rate based on RECIST criteria, the *g*‐score captures tumor growth and also incorporates time in characterizing tumor dynamics.

Trastuzumab deruxtecan (T‐DXd) became the standard of care for second‐line treatment of advanced HER2‐positive and HER2‐low (IHC 1 + or IHC 2+/ISH‐) breast cancers^27^ based on phase 3 confirmatory trials demonstrating significant advantages in overall survival (OS) and progression free survival (PFS) compared to trastuzumab emtansine in DESTINY‐Breast03 trial^28^ and to therapy of physician’s choice (TPC) in DESTINY‐Breast04 trial.^29^ The past decades have seen the testing of a large number of targeted therapies and novel agents in metastatic breast cancer, a diverse disease that consists of many subtypes.^30^ It took tremendous effort and cost to identify the very few efficacious agents like trastuzumab deruxtecan able to improve survival. Novel approaches are needed to screen agents early in clinical development and to inform decisions more accurately. In this study, we applied this approach to the two randomized phase 3 studies (DB-03 and DB-04). Our objectives were to characterize the impact of trastuzumab deruxtecan on tumor growth during treatment and to evaluate the utility of the tumor growth rate model to inform treatment efficacy for future clinical trials.

## Methods

### *g*-score Model

**Figure 1.**
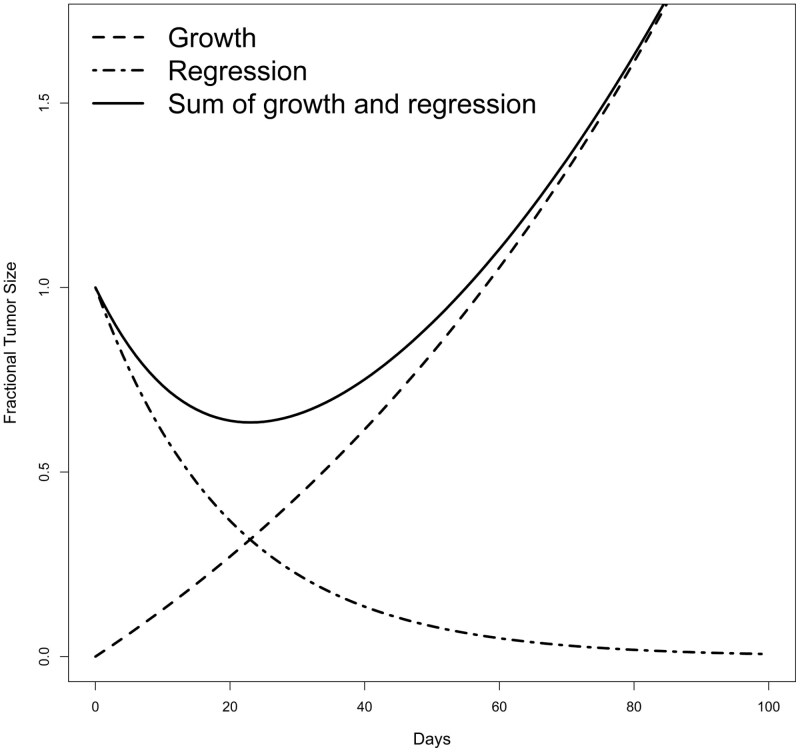
Illustration of g-score modeling from Stein et al (2008)^9^.


The *g*‐score model^4^ utilizes tumor burden measurements obtained through radiographic assessments during treatment. As shown in Figure 1, this model assumes 2 independent but simultaneous processes^9^: exponential decay or regression of the tumor at rate *d*, and exponential growth or regrowth at rate *g*. With sufficient tumor assessment data, most patients can be categorized into one of the four models below:

$$\begin{array}{l} gd\;:\;f\left(t\right)\;=\;e^{-dt}\;+\;e^{gt}-1 \end{array}$$ (1)

$$\begin{array}{l} gx\;:\;f\left(t\right)\;=\;e^{gt} \end{array}$$ (2)

$$\begin{array}{l} dx\;:\;f\left(t\right)\;=\;e^{-dt} \end{array}$$ (3)

$$\begin{array}{l} gd\phi \;:\;f\left(t\right)\;=\;\phi \;e^{-dt}\;+\;\left(1\;\phi \right)\;e^{gt} \end{array}$$ (4)

where f(t) denotes the tumor burden at time t (in days), normalized to the radiographic measurements at t = 0. The rate of regression or decay is denoted by d (in day^–1^). ϕ represents the treatment‐sensitive fraction of the tumor, while (1ϕ) indicates the fraction that is either absolutely or relatively resistant to treatment. For each patient, the tumor’s trajectory will be fitted to these 4 models by non‐linear regression and the model selection will be conducted based on the Akaike information criterion (AIC)^31^ and each parameter stays in the model when the p value is < 0.1. A patient is determined as “not fit” to any of the 4 models if all parameters have their p values greater than 0.1 in all models. The gd model in equation (1) indicates a mixture of tumor decay and growth, which is most common. When d is not detected, the gd model (equation (2)) is reduced to the gx model, and the tumor growth rate is estimated using the gx equation. The gx model reflects the data showing continuous growth in tumor burden from the initiation of treatment. When g is not detected, the gd model is reduced to the dx model (3), and the rate of tumor decay is estimated using the dx equation. The dx model indicates continuous reduction in tumor burden from the start of treatment with growth not detectable with the data acquired before database lock or before treatment discontinuation, so mathematically dx patients have a g‐score of 0. The gdϕ model in equation (4) includes an additional parameter ϕ, representing the proportion of tumor that is sensitive to treatment. In this model, d denotes the decay rate of the treatment sensitive portion of the tumor, while g represents the growth rate of the treatment resistant portion. The gdϕ model is needed to fit data that includes a prolonged nadir before growth begins. Although the tumor trajectory is modeled by both growth and decay patterns simultaneously, only the growth rate is extracted as the g‐score. The tumor growth rate is mathematically related to tumor doubling time,^32^ allowing one to estimate tumor growth as tumor doubling time by dividing ln(2) by g, or 0.693/ g.

### Data sources

The data for this analysis came from 2 randomized, open‐label, phase 3 trials: DESTINY‐Breast3 (DB-03)^33^ and DESTINY‐Breast04 (DB-04).^34^ The DB-03 study enrolled 524 patients with confirmed HER2‐positive unresectable or metastatic breast cancer between July 20, 2018, and June 23, 2020, with database cutoff date of May 21, 2021. These patients had either previously received trastuzumab and taxane therapy for metastatic disease or experienced disease recurrence during or within 6 months of completing adjuvant therapy, which included trastuzumab and taxane. Patients with asymptomatic previously treated and untreated brain metastases were also eligible for enrollment.^35^ Patients were stratified based on hormone receptor status (positive or negative), prior pertuzumab treatment, and history of visceral disease. They were then randomly assigned in a 1:1 ratio to receive either T‐DXd 5.4 mg/kg or T‐DM1 3.6 mg/kg administered intravenously every 3 weeks.^33^

The DB-04 study randomized 557 patients with hormone receptor positive or negative metastatic breast cancer and centrally confirmed HER2‐low expression, enrolled between December 27, 2018, and December 31, 2021, with a database cutoff date of Jan 11, 2022. These patients had previously received 1 or 2 lines of chemotherapy or experienced disease recurrence during or within 6 months after completing adjuvant chemotherapy and patients with HR + disease had received at least one line of endocrine therapy. Patients were randomly assigned in a 2:1 ratio to receive T‐DXd 5.4 mg/kg, administered intravenously every 3 weeks, or TPC. Randomization was stratified based on HER2‐low status (IHC 1+ or IHC 2+ and ISH−), the number of previous lines of chemotherapy for metastatic disease (1 or 2), and HR status (positive with previous CDK4/6 inhibitor therapy, positive without previous CDK4/6 inhibitor therapy, or negative).^34^ For both studies, tumor assessments were scheduled every 6 weeks (± 7 days) from randomization until disease progression independent of treatment cycle. A CT and/or MRI (CT or MRI with ≤5 mm cuts) of chest, abdomen, and pelvis was to be used for tumor assessment unless another modality of disease assessment was necessary for the lesions. The same assessment modality was to be used throughout the study for all assessments for each subject unless prior approval was obtained from Sponsor or designee. Unscheduled tumor assessments could be performed if progression was suspected. A CT or MRI of the brain was mandatory for all subjects included with baseline stable brain metastases. The tumor measurement data used in this analysis were assessed by blinded independent central review (BICR).

## Results

**Figure 2.**
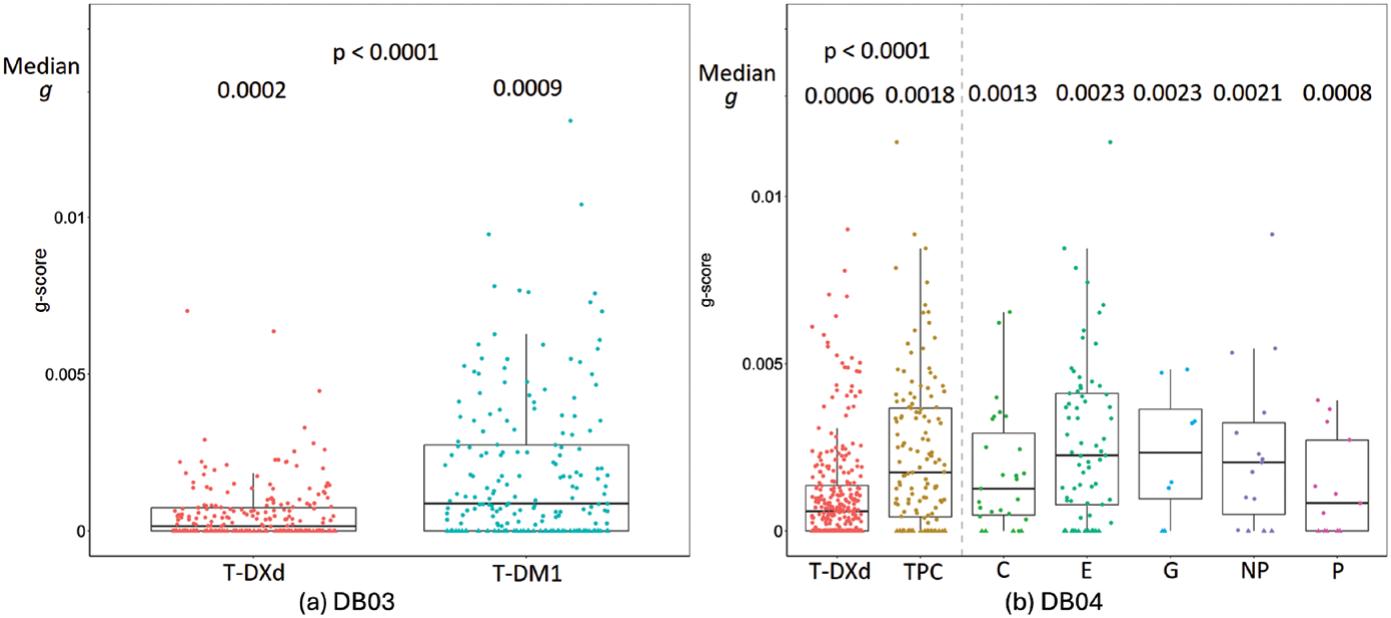
T‐DXd showed significant superiority in reducing tumor growth (g) in both DB-03 (a) and DB-04 (b) studies. C: Capecitabine. E: Eribulin. G: Gemcitabine. NP: Nab-Paclitaxel. P: Paclitaxel.

Table 1 shows the classification of subjects based on tumor growth characteristics, including gx, gd, gdϕ, and dx. In the DB-03 study, 190 (82%) subjects in the T‐DM1 treatment group and 241 (98%) subjects in the T‐DXd treatment group had tumor trajectories that fit one of the four g‐score models. A g-score was not assessed in 19 (8%) subjects in the T‐DM1 group and no subjects in the T‐DXd group who had only one post-baseline assessment with less than a 20% change from baseline. The 20% threshold was selected to align with the RECIST definition of progression when only one post‐baseline tumor assessment is available. The T‐DXd treatment group showed a significantly higher percentage whose data fit the regression‐only dx model compared to the T‐DM1 treatment group (48% vs 23%) and a lower percentage with data best described by the growth‐only model gx (2% vs 16%). The g‐score in the T‐DXd arm was significantly lower than that in the T‐DM1 arm with median *g* (IQR) (0.02(0 − 0.07) *× *10^–2^ vs 0.09(0 − 0.27) *× *10^–2^, *P < *.0001, respectively). Within subjects who had tumor growth (*g *> 0), the median *g* for T‐DXd and T‐DM1 were 0.07 *× *10^–2^ and 0.18 *× *10^–2^ with the corresponding tumor doubling times (TDT)^32^ of 977 days vs 388 days respectively. When the requirement of at least 20% change for subjects with only one on-treatment tumor assessment is removed, changes in the model distribution and median *g* estimates were minimal (Supplementary Table S2). The distribution of *g*‐score is displayed by the treatment arm (Figure 2) and key prognostic factors (Supplementary Figure S1) for the DB-03 study. Supplementary Figure S2 shows the classification of subjects based on tumor growth characteristics by key prognostic factors. T‐DXd demonstrated a consistent trend in reducing tumor growth (*g*) across key prognostic factors.

**Table 1.** Classification of subjects by g-score models.

	DB-03		DB-04	
	T‐DM1 (*n* = 232)	T‐DXd (*n* = 245)	TPC (*n* = 162)	T‐DXd (*n* = 348)
Fit to a model (*dx*/*gd*/*gd*ϕ/*gx*), n%	190 (82%)	241 (98%)	132 (81%)	316 (91%)
dx, n(%)	54 (23%)	117 (48%)	28 (17%)	113 (32%)
gd, n%	78 (34%)	106 (43%)	57 (35%)	140 (40%)
gdϕ, n%	22 (9%)	12 (5%)	12 (7%)	36 (10%)
gx, n(%)	36 (16%)	6 (2%)	35 (22%)	27 (8%)
Not fit to a model	23 (10%)	4 (2%)	17 (10%)	20 (6%)
Only 1 post baseline but < 20% change	19 (8%)	0 (0%)	13 (8%)	12 (3%)
Median *g* (IQR) (*× *10^–2^/day)^a^	0.09 (0.00‐0.27)	0.02 (0.00‐0.07)	0.18 (0.04‐0.37)	0.06 (0.00‐0.14)
* P* *^b^*	P<0.0001		P<0.0001	
Subjects with tumor growth (gx, gd, gdϕ)				
Median *g* (IQR) (*× *10^–2^/day)	0.18 (0.08‐0.38)	0.07 (0.04‐0.14)	0.25 (0.13‐0.40)	0.10 (0.06‐0.21)
Median TDT(IQR) (*× *100 days)	3.88 (1.84‐8.55)	9.77 (5.09‐16.49)	2.74 (1.72‐5.43)	6.66 (3.27‐11.05)

*Note*: Only evaluable subjects are included in the analysis who had tumor trajectories in sum of diameters of baseline and at least 1 post baseline assessment by blinded independent central review. TDT = tumor doubling time, calculated as *ln*(2)/*g* for *g *> 0.

^a^Median *g* is calculated based on subjects whose tumor trajectories can fit to a model.

^b^Mann‐Whitney test.

In the DB-04 study, 132 (81%) subjects in the TPC treatment group and 316 (91%) subjects in the T‐DXd treatment group had tumor trajectories that were fit to 1 of the 4 g‐score models. In addition, 13 (8%) subjects in the TPC group and 12 (3%) subjects in the T‐DXd group had only one post‐baseline assessment with less than a 20% change from baseline, and no g‐score model could be fitted for the data from these subjects. The T‐DXd treatment group showed a higher percentage of data best fit by the regression‐only model compared to the TPC treatment group (32% vs. 17%), and a lower percentage of data best described by the growth‐only model (8% vs. 22%). The g‐score in the T‐DXd arm was significantly lower than that in the TPC arm with median *g* (IQR) (0.06(0 − 0.14) *× *10^–2^ vs 0.18(0.04 − 0.37) *× *10^–2^, *P < *.0001). Within subjects who had tumor growth (*g *> 0), the median *g* for T‐DXd and TPC were 0.10 *× *10^–2^ and 0.25 *× *10^–2^ with corresponding tumor doubling times of 666 days vs 274 days respectively. The distribution of g‐scores by treatment arm (Figure 2) and key prognostic factors (Supplementary Figure S3) in the DB-04 study. For the DB-04 study, the g‐score distribution is also shown for each therapy of physician’s choices. Supplementary Figure S4 shows the classification of subjects based on tumor growth characteristics by key prognostic factors. Similar to the observations in DB-03 study, T‐DXd demonstrated a consistent trend in reducing tumor growth(*g*) across the prognostic factors in DB-04 in the HER2‐low pretreated breast cancer population.

**Figure 3.**
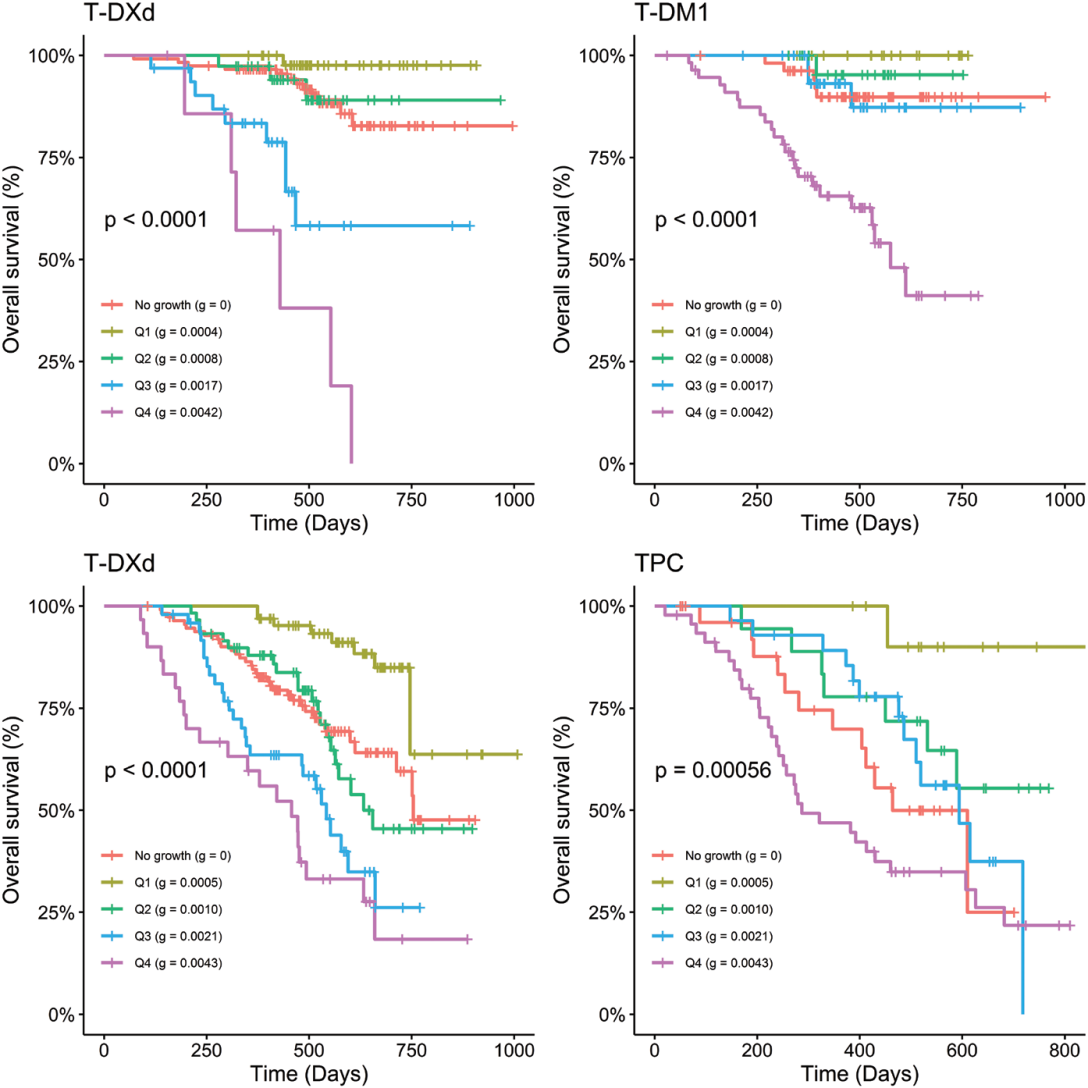
*g* is highly associated with OS in DB-03 (top row) and DB-04 (bottom row) studies. No growth: dx subjects; Q1‐Q4: quartiles of *g* are calculated for subjects pooled from 2 treatment groups. In each quartile group, the median *g* is calculated: quartile (median *g*).

**Figure 4.**
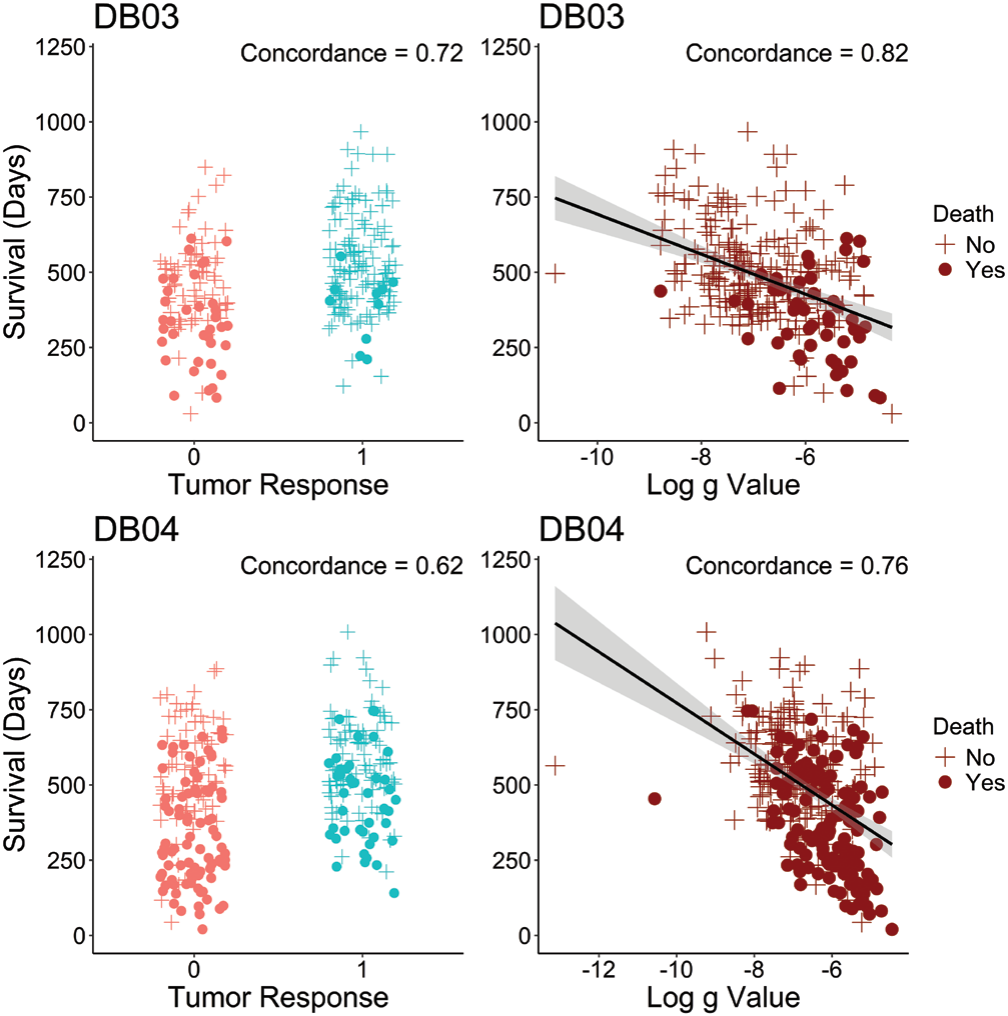
*g* has improved concordance with survival compared to tumor response in DB-03 (top row) and DB-04 (bottom row) studies among subjects with tumor growth (g>0). The concordance is calculated based on Cox regression models.

The association between tumor growth rate(*g*) and overall survival was explored using Kaplan‐Meier plots and Cox regression methods. Figure 3 presents Kaplan‐Meier plots of OS by g quartiles and dx subjects (no tumor growth detected) treated with T‐DXd, T‐DM1, and TPC in the DB-03 and DB-04 studies. The first quartile (Q1) corresponds to subjects with the lowest *g*, indicating the slowest tumor growth, while the fourth quartile (Q4) represents those with the highest g‐scores, reflecting the fastest growth. In this retrospective analysis, a significant association was observed between *g* and OS for subjects treated with T‐DXd, T‐ DM1, and TPC in both studies. A general monotonic trend was noted across quartiles Q1, Q2, Q3, and Q4. Interestingly, OS in patients whose tumor best fit the dx model was comparable to the OS of Q1 subjects. A strong association between g  and PFS was also observed, as shown in Supplementary Figure S5. In addition to the Kaplan‐Meier plots, hazard ratios for OS (Table 2) and PFS (Supplementary Table S1) were estimated using Cox regression models for dx, Q1, Q2, and Q3 compared to Q4 by treatment group in both studies. A consistently monotonic trend was observed in hazard ratios for Q1, Q2, and Q3 compared to Q4 for both OS and PFS. For example, in the DB-03 study, the OS hazard ratios in the T‐DXd treatment group were 0.01, 0.07, and 0.36 for Q1, Q2, and Q3, while in the T‐DM1 treatment group, they were 0.0, 0.07, and 0.18, respectively. Similarly, in the DB-04 study, the OS hazard ratios in the T‐DXd treatment group were 0.1, 0.34, and 0.66, while in the TPC treatment group, they were 0.07, 0.33, and 0.46. PFS analysis showed similar results. Additionally, the median PFS values for the 4 quartiles show a consistent, monotonic decrease from Q1 to Q4 across treatment groups in both studies. Since the OS medians were not reached in most quartiles, the 1‐year OS rate is used to illustrate the differences. Similarly, the 1‐year OS rate also exhibits a monotonic decline from Q1 to Q4 across treatment groups in both studies. In addition, the predictive value of the g-score and tumor response for overall survival is assessed in terms of concordance using Cox regression models. Figure 4 demonstrates an improved concordance for the g-score as a predictor of survival compared to tumor response, with an increase from 0.72 to 0.82 in DB-03 study and from 0.62 to 0.76 in DB-04 study.

**Table 2.** OS analysis by g‐score quartiles.

DB-03	T‐DXd			T‐DM1		
	N	1‐year OS (%)	OS HR (95%CI)	N	1‐year OS (%)	OS HR (95%CI)
dx	117	96.6	0.08 (0.03‐0.21)	54	96.2	0.17 (0.07‐0.45)
Q1	46	100	0.01 (0‐0.11)	19	100	0.0 (0‐Inf)
Q2	38	97.4	0.07 (0.02‐0.28)	27	100	0.07 (0.01‐0.53)
Q3	32	83.4	0.36 (0.13‐1.02)	33	100	0.18 (0.05‐0.59)
Q4	8	57.1	Ref	57	70.3	Ref
**DB-04**	**T‐DXd**			**TPC**		
	*N*	1‐year OS (%)	OS HR (95%CI)	*N*	1‐year OS (%)	OS HR (95%CI)
dx	113	84.5	0.31 (0.18‐0.53)	28	69.8	0.62 (0.32‐1.21)
Q1	65	100	0.1 (0.05‐0.22)	12	100	0.07 (0.01‐0.48)
Q2	59	87.9	0.34 (0.19‐0.62)	18	77.8	0.33 (0.14‐0.75)
Q3	49	63.6	0.66 (0.37‐1.17)	28	89.1	0.46 (0.24‐0.89)
Q4	30	59.6	Ref	46	46.9	Ref

*Note*: Subjects are categorized to the 4 quartiles Q1, Q2, Q3, and Q4 based on the thresholds determined from the pooled data in each study. T‐DXd treatment group had greater percentages of subjects in Q1 and Q2 due to its g‐score distribution shift to smaller g‐score (better) than the control arm in each study. TPC = treatment of physician’s choice.

**Figure 5.**
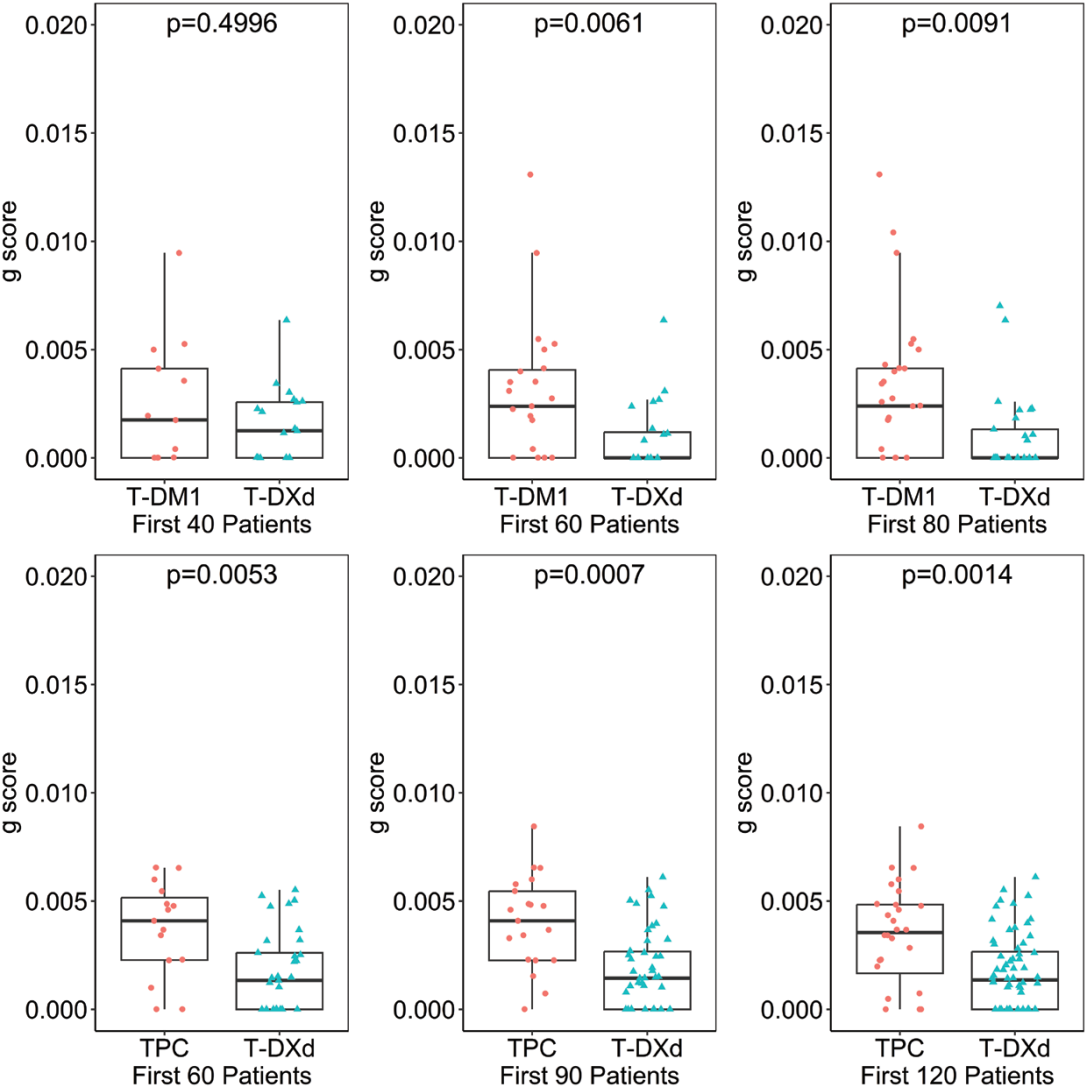
T‐DXd’s improvement in g‐score can be demonstrated with as few as first 60 subjects, provided the 60th subject had been followed for 14 weeks in DB-03 (top) and DB-04 (bottom) studies. In DB-03 study (1:1 randomization ratio), the analyses were based on first randomized 40, 60, and 80 subjects when the 40th, 60th, and 80th subject followed for 14 weeks respectively; in DB-04 study (2:1 randomization ratio), the analyses were based on first randomized 60, 90, and 120 subjects.

As a reference, the similar quartile analysis for parameter d was also performed and included in Supplementary Figure S6 and Supplementary Figure S7. As reported previously,^4,9,17,23^d appears less correlated with OS and PFS, and at present its value if any in assessing efficacy and informing decisions remain uncertain. One major advantage of the g‐score is its early availability compared to survival data.**.**Supplementary Figure S8 illustrates the g‐score distribution estimated with an increasing number of tumor scans in DB-03 and DB-04 studies. While the experimental arm showed improvement in g‐score compared to the control group with only two post‐baseline scans (3 points), the g‐scores obtained at this early stage in the T‐DXd arm may not align with the values observed when more scans became available. Figure 5 further depicts the g‐score by treatment arm when the 60th, 90th, and 120th subjects had been followed for 14 weeks since randomization. In this analysis, subjects enrolled earlier had potentially more scans analyzed, reflecting the real‐world study analyses at different data cutoffs. T‐DXd’s improvement in g‐score can be demonstrated with as few as the first 60 subjects, provided the 60th subject had been followed for 14 weeks.

## Discussion

g‐score has been reported to have a strong correlation with survival in multiple tumor types and with diverse therapeutics. This is the first report assessing g‐score in tumors treated with ADCs and its association with PFS and OS based on 2 large phase 3 randomized clinical trials in metastatic breast cancer. Our findings are consistent with previous reports finding g‐score strongly associated with both PFS and OS data. Our analysis demonstrates the superiority of T‐DXd in reducing the tumor growth rate (*g*) compared to T‐DM1 in the DB-03 study and compared to therapy of physician’s choice (TPC) in the DB-04 study for pre‐treated metastatic breast cancer. In addition, the treatment effect of T‐DXd compared to the control treatments could be demonstrated with a much smaller sample size at an earlier time of study conduct in both studies. Compared to tumor response rate which only categorizes patients according to the best shrinkage, g‐score is an early intermediate endpoint that incorporates the dimension of time in addition to size in assessing efficacy. g‐score represents a new tool that can provide unique perspectives in interpreting clinical data for decision making in early-phase studies, such as for dose selection and for population selection, and for interim analysis of results in late-phase studies. It may also be useful in pipeline prioritization based on multiple early phase studies with limited data, where examination of the totality of data available is essential for evaluation including safety, efficacy, biomarkers, and PK/PD endpoints.^36^ Decision‐making in this context can be notably complex, especially when the sample size is limited and confounded by challenges such as heterogeneous disease characteristics, varying biomarker expression levels, and the lack of appropriate controls. Additionally, objective response based on imaging criteria, such as Response Evaluation Criteria in Solid Tumors (RECIST)^37,38^ and Response Assessment in Neuro‐Oncology (RANO)^39^ is commonly used to assess tumor response; however, the prognostic significance of ORR can vary across different tumor types and therapeutic agents. With an observed strong correlation with long-term survival endpoints, g‐score can support earlier and improved decision making.

The current analyses were based on a one‐dimensional sum of diameters from existing study data. Other studies have shown that growth rates based on volumetric tumor burden demonstrate excellent performance characteristics.^13,40,41^ Further exploration of volumetric g‐scores could provide valuable insights into tumor dynamics. One technical consideration is the interpretation of dx  subjects who had no detectable tumor growth (g = 0) based on available data and whose data is best described by the dx equation, but whose survival was similar to that of subjects comprising the slower quartiles (Q1 / Q2) of g. This suggests that the dx cohort in patients with metastatic disease may represent a range of clinical situations including patients who discontinued treatment despite having had only regression of tumor and in whom the tumor growth rate after discontinuation could have better described the survival outcome.

Since existing response criteria do not take into account individual tumor growth kinetics before the initiation of experimental therapies, distinguishing stable disease as a result of the natural progression of the disease or therapy‐induced cytostasis becomes challenging. One approach to assessing treatment benefit is to understand a more complete tumor trajectory, using historical scans available before trial enrollment when receiving prior anticancer therapies. By comparing to the historical trajectory, additional insights may be obtained that uncover whether the experimental therapy can potentially alter the trajectory of tumor progression in patients receiving study treatment.^42^ In metastatic prostate cancer, patients whose g rate was slower with olaparib compared to the g rate on the previous treatments achieved significantly better survival than those whose g rate was not slower with olaparib.^43^ We were unable to perform such analysis in DB-03 and DB-04 studies because prior scans were not collected. Such analysis evaluating g rates within each subject as they are treated with different agents can be particularly useful in single-arm studies and small randomized studies where confounding effects cannot be reliably controlled through randomization.

In addition to CT and MRI radiographic assessments, surrogate blood markers such as prostate‐specific antigen (PSA) for prostate cancer,^21^ CA19‐9 for pancreatic cancer,^4^ and CA‐125 for ovarian cancer^16^ have been used for g‐score modeling. The derived g‐score was found significantly associated with survival in those settings as well. Since g‐ g-score modeling incorporates the dimension of time, this modeling approach requires subjects to have both a baseline assessment and at least one post‐baseline assessment. If a subject lacks adequate post‐baseline assessments, a g‐score cannot be generated. As a result, those subjects’ efficacy should be evaluated using alternative efficacy endpoints and taking their characteristics into account. To further understand the association between g‐score and survival endpoints at trial‐level, we are performing ongoing evaluation of g‐score’s predictive value in a large‐scale meta‐analysis based on multiple large, randomized phase 3 studies. When the requirement of at least 20% change for subjects with only one on-treatment tumor assessment is removed, the changes in the distribution of classification and median g estimates are minimal for each treatment group. These subjects are either modeled as growth only or decay only. However, based on Supplementary Figure S8, the g-score obtained based on only one on-treatment tumor assessment may not provide a reliable estimate of the g-score.

## Supplementary Material

oyaf057_suppl_Supplementary_Tables_1-2

oyaf057_suppl_Supplementary_Figures_1-8

## Data Availability

Anonymized individual participant data (IPD) on completed studies and applicable supporting clinical study documents may be available upon request at https://vivli.org/. In cases where clinical study data and supporting documents are provided pursuant to our company policies and procedures, Daiichi Sankyo Companies will continue to protect the privacy of company and our clinical study subjects. Details on data sharing criteria and the procedure for requesting access can be found at this web address: https://vivli.org/ourmember/daiichi-sankyo/.
