# Supplementary material for: Correlation between tumor growth rate and survival in patients with metastatic breast cancer treated with trastuzumab deruxtecan
Source: Oncologist. 2025 May 11;30(5):oyaf057. doi: 10.1093/oncolo/oyaf057 (PMC12065935; doi:10.1093/oncolo/oyaf057)
Supplement: oyaf057_suppl_Supplementary_Tables_1-2 [file oyaf057_suppl_supplementary_tables_1-2.docx]

Supplementary Table 1. PFS analysis by g‐score quartiles

| **DB-03** | **T‐DXd** | | | **T‐DM1** | | |
| --- | --- | --- | --- | --- | --- | --- |
|  | N | Median | HR (95%CI) | N | Median | HR (95%CI) |
| dx | 117 | NR | 0.02  (0.01-0.05) | 54 | Not Reached | 0.04  (0.02-0.08) |
| Q1 | 46 | NR | 0.01  (0-0.04) | 19 | Not Reached | 0.04  (0.02-0.1) |
| Q2 | 38 | 16.8 | 0.03  (0.01-0.1) | 27 | 14 | 0.06  (0.03-0.13) |
| Q3 | 32 | 8.3 | 0.19  (0.08-0.5) | 33 | 6.8 | 0.28  (0.16-0.46) |
| Q4 | 8 | 4.1 | Ref | 57 | 2.7 | Ref |
|  |  |  |  |  |  |  |
| **DB-04** | **T‐DXd** | | | **TPC** | | |
|  | N | PFS  Median | PFS HR (95%CI) | N | PFS  Median | PFS HR (95%CI) |
| dx | 113 | 17.9 | 0.02  (0.01-0.03) | 28 | 8.5 | 0.07 (0.03-0.15) |
| Q1 | 65 | 18.8 | 0.01  (0.01-0.03) | 12 | 12.8 | 0.04 (0.01-0.1) |
| Q2 | 59 | 9.6 | 0.05  (0.03-0.08) | 18 | 13.9 | 0.06 (0.03-0.13) |
| Q3 | 49 | 6.9 | 0.12  (0.07-0.2) | 28 | 7.1 | 0.2 (0.11-0.36) |
| Q4 | 30 | 2.6 | Ref | 46 | 2 | Ref |

Note: Subjects are categorized to the four quartiles Q1, Q2, Q3 and Q4 based on the thresholds determined from the pooled data in each study. T‐DXd treatment group had greater percentages of subjects in Q1 and Q2 due to its g‐score distribution shift to smaller g‐score (better) than the control arm in each study. TPC = treatment of physician’s choice

Supplementary Table 2. Classification of subjects by g-score models without excluding subjects having only one on-treatment scan but within 20% change from baseline

|  | **DB-03** | | **DB-04** | |
| --- | --- | --- | --- | --- |
|  | **T‐DM1 (n=232)** | **T‐DXd (n=245)** | **TPC (n=162)** | **T‐DXd (n=348)** |
| Fit to a model (*dx*/*gd*/*gdϕ*/*gx*), n% | 207 (89%) | 241 (98%) | 145 (90%) | 328 (94%) |
| $dx, n(\%)$ | 59 (25%) | 117 (48%) | 33 (20%) | 117 (34%) |
| $gd, n\%$ | 78 (34%) | 106 (43%) | 57 (35%) | 140 (40%) |
| $gd\phi, n\%$ | 22 (9%) | 12 (5%) | 12 (7%) | 36 (10%) |
| $gx, n(\%)$ | 48 (21%) | 6 (2%) | 43 (27%) | 35 (10%) |
| Not fit to a model | 25 (11%) | 4 (2%) | 17 (10%) | 20 (6%) |
| Median *g* (IQR) (*×*10^–2^/day)^a^ | 0.09 (0.00‐0.27) | 0.02 (0.00‐0.07) | 0.17 (0.03‐0.35) | 0.06 (0.00‐0.14) |
| *P^b^* | $p<0.0001$ | | $p<0.0001$ | |
| Subjects with tumor growth ($gx$, $gd$, $gd\phi$) |  |  |  |  |
| Median *g* (IQR) (*×*10^–2^/day) | 0.18 (0.08‐0.37) | 0.07 (0.04‐0.14) | 0.25 (0.12‐0.39) | 0.11 (0.06‐0.21) |
| Median TDT(IQR) ( *×*100 days) | 3.90 (1.89‐8.67) | 9.77 (5.09‐16.49) | 2.74 (1.77‐5.67) | 6.55 (3.29‐10.99) |
