# Supplementary material for: Correlation between tumor growth rate and survival in patients with metastatic breast cancer treated with trastuzumab deruxtecan
Source: Oncologist. 2025 May 11;30(5):oyaf057. doi: 10.1093/oncolo/oyaf057 (PMC12065935; doi:10.1093/oncolo/oyaf057)
Supplement: oyaf057_suppl_Supplementary_Figures_1-8 [file oyaf057_suppl_supplementary_figures_1-8.docx]

Supplementary Materials





Supplementary Figure 1. T‐DXd showed consistent effect in reducing tumor growth (g) across prognostic factors in DB-03 study. *: $p < 0.01$, **: $p < 0.001$, ***:$p < 0.0001$





Supplementary Figure 2. Classification of subjects by g‐score modeling and prognostic factors in DB-03 study. T‐DXd shows less proportion of subjects having *gx* and more proportion of subjects having *dx* compared to T‐DM1 across the prognostic factors.








Supplementary Figure 3. Classification of patients by g‐score modeling and prognostic factors in DB-04 study. T‐DXd shows less proportion of subjects having *gx* and more proportion of subjects having *dx* compared to TPC across the prognostic factors.








Supplementary Figure 4. T‐DXd showed consistent effect in reducing tumor growth (g) across prognostic factors in DB-04 study. *: p < 0.01, **: p < 0.001, ***: p < 0.0001





Supplementary Figure 5. g is highly associated with PFS in DB-03 (top row) and DB-04 (bottom row) studies. No growth: dx subjects; Q1‐Q4: quartiles of g are calculated for subjects pooled from two treatment groups. In each quartile group, the median g is calculated: quartile (median g).





Supplementary Figure 6. Assessment of association between *d* and OS in DB-03 (top row) and DB-04 (bottom row) studies by Kaplan‐Meier plots. The association between *d* and OS is not as strong as the association between *g* and OS. No decay: *gx* subjects; Q1‐Q4: quartiles of d are calculated for subjects pooled from two treatment groups. In each quartile group, the median g is calculated: quartile (median g).





Supplementary Figure 7. Assessment of association between *d* and PFS in DB-03 (top row) and DB-04 (bottom row) studies by Kaplan‐Meier plots. The association between *d* and PFS is not as strong as the association between *g* and OS. No decay: *gx* subjects; Q1‐Q4: quartiles of g are calculated for subjects pooled from two treatment groups. In each quartile group, the median g is calculated: quartile (median g).








Supplementary Figure 8. T‐DXd demonstrated superior tumor growth reduction (g) early in both the DB-03 (top) and DB-04 (top) studies. The g‐score distribution for each treatment group was more stabilized when three or more post‐baseline scans (four or more data points) were available. In each comparison, the median (min‐max) of time point tumor assessments (points) and follow up in months are provided. P value is calculated based on Mann‐Whitney test.
